# Therapeutic Notes

**Published:** 1896-03-07

**Authors:** 


### Therapeutic Notes.

### ALKALOIDS OF IPECACUANHA.

Ipecacuanha sine emetine has for some time past been obtainable as a commercial preparation, and, with a certain section of the profession, it has had a more or less extensive use in dysenteric conditions. Doubtless it would have been in still greater favour for this purpose could it have been depended upon as entirely emetine-free, and had our knowledge been more exact as to the nature of the principle in ipecacuanha which exercises a specific influence in dysentery. The recent researches of Dr. B. H. Paul and Mr. A. J. Cownley have done much to clear up our ignorance as to the chemical composition of crude ipecacuanha, and have brought to light the fact that there is, at least, one other alkaloid besides emetine, and probably a second. Armed with this further knowledge, exact information as to the therapeutic action of the different constituents is merely a matter of time; and having found that which is specific in dysentery, we shall be able to treat that disorder in a scientific and accurate manner. The new alkaloid which has been discovered by the above observers has been called cephæline, and it appears that it contains one methyl group ($CH_2$) less than the first-discovered alkaloid, consequently it has been assumed that emetine is methyl-cephæline. The chemical properties of the two bodies may be compared as follows:—

| Emetine. | Cephæline. |
|---|---|
| $C_{13} H_{22} No_2$ | $C_{14} H_{20} No_2$ |
| Uncrystallisable. | Crystallisable. |
| Forms crystalline salts. | Forms crystalline salts. |
| Fuses at 68 deg. C. | Fuses at 102 deg. C. |
| Readily soluble in ether, alcohol, and chloroform. | Not so soluble in ether, &c., as emetine. |
| Insoluble in alkalies. | Soluble in alkalies. |

The therapeutic actions of the two bodies appears very similar in nature, though differing in certain respects. Emetine has less influence on the vomiting centre and on the motor nerve endings; on the other hand, the methyl compound has a more powerful local action as a vaso-constrictor. Cephæline appears to be a reliable emetic in doses of $\frac{1}{12}$—$\frac{1}{6}$ gr.; its action is, however, somewhat slow. As an expectorant in acute catarrhal conditions, &c., where emesis is not desired, emetine in small doses of $\frac{1}{12}$ gr. seems well adapted.

The third alkaloid, if there really be such present in ipecacuanha, must differ in physical rather than in chemical properties from the two defined bodies above described. There appears to be good ground for believing that ipecacuanha deprived of its alkaloidal constituents is as efficacious as the crude drug. Willigk has supposed this specific action to be due to ipecacuanhic acid. Messrs. Paul and Cownley have repeated his experiments, and show that the body which he believed to be ipecacuanhic acid is, in reality, a mixture, and hence his results must be inconclusive. Further results of their researches show that cartagena root (New Granada ipecacuanha) contains a larger proportion of the more active alkaloid cephæline than does the ordinary Brazilian variety. Hence preparations made from the former are stronger than those made from the latter. For this reason the ordinary preparations of ipecacuanha must differ considerably as to strength according as they are prepared from one or other of these roots; and further than this, all preparations of ipecacuanha as at present existing in our Pharmacopœia undergo chemical changes on keeping.

Pharm. Journal, liii. 61, and liv. 373.

